# Indicators for dental appointment scheduling in primary health care: a national cross-sectional study

**DOI:** 10.1186/s12889-021-12319-x

**Published:** 2021-12-08

**Authors:** Estêvão Azevedo Melo, Livia Fernandes Probst, Luciane Miranda Guerra, Elaine Pereira da Silva Tagliaferro, Alessandro Diogo De-Carli, Antonio Carlos Pereira

**Affiliations:** 1 Piracicaba Dental School, State University of Campinas (UNICAMP), Piracicaba, Brazil; 2 Health Technology Assessment Unit, Hospital Alemão Oswaldo Cruz (HAOC), São Paulo, Brazil; 3 School of Dentistry, São Paulo State University (UNESP), Araraquara, Brazil; 4 Faculty of Dentistry, Federal University of Mato Grosso do Sul (UFMS), Campo Grande, Brazil

**Keywords:** Public health practice, Dental health services, Public health dentistry, Public health systems research, Community dentistry

## Abstract

**Background:**

Integrated dental services within the Health System, particularly at primary health care, are crucial to reverse the current impact of oral diseases, which are among the most prevalent diseases worldwide. However, the use of dental services is determined by complex phenomena related to the individual, the environment and practices in which care is offered. Therefore, factors associated with dental appointments scheduling can affect positively or negatively the use of dental services. The aim of the present study was to evaluate the indicators for dental appointment scheduling in Primary Health Care (PHC).

**Methods:**

The present is a cross-sectional analytical study that used data from the external assessment of the third cycle of the National Program for Improving Access and Quality in Primary Care (PMAQ-AB), carried out between 2017 and 2018, in Brazil. The final sample consisted of 85,231 patients and 22,475 Oral Health teams (OHTs). The outcome variable was the fact that the user sought for a dental appointment at the Primary Health Care Unit. A multilevel analysis was carried out to verify the association between individual variables (related to users) and contextual variables (related to the OHTs) in relation to the outcome.

**Results:**

Only 58.1% of the users interviewed at these Primary Health Care Units seek the available dental care. The variables with the greatest effect on the outcome were the patient’s age up to 42 years old (OR = 2.03, 95% CI: 1.96–2.10), at individual level, and ‘oral health teams that assisted no more than a single family health team (FHT)’ (OR = 1.29, 95% CI: 1.23–1.36) at contextual level. Other variables were also associated with the outcome, but with a smaller effect size.

**Conclusion:**

In conclusion, users’ age and work process of OHT were indicators for dental appointment scheduling. Our results suggest that when OHT put the National Oral Health Policy guidelines into practice, by assisting only one FHT, the chance for PHC users seeking dental appointments is higher than OHTs that assist more than one FHT. Regarding age, patients aged up to 42 years are more likely to seek an appointment with a dentist.

**Supplementary Information:**

The online version contains supplementary material available at 10.1186/s12889-021-12319-x.

## Background

Oral diseases are among the most prevalent ones worldwide and, although they are largely preventable, public oral health policies remain inadequately funded for disease prevention and treatment [1]. To reverse this situation, it is necessary for dental services to act in an integrated manner within the public Health Care System, particularly with primary health services [1–3].

However, even in scenarios with guaranteed and subsidized care, the use of dental services can be influenced by issues related to the individual, such as overall awareness of oral health or a previous negative experience [4]. In addition to individual issues, the use of available health services is determined by complex interactions with the environment and with the practices in which care is offered [5].

After the creation of Unified Health System (SUS, *Sistema Único de Saúde*) in 1988, and National Oral Health Policy (PNSB, *Política Nacional de Saúde Bucal*) in 2004, important changes in health and oral health care have been achieved in Brazil [6, 7]. By implementing a new model of care and establishing specific regulations for planning, financing, governance, and provision of healthcare services, SUS has brought nearly universal access to health care and has kept track of the population’s health needs [8, 9].

SUS model of care in Primary Health Care includes multiprofessional teams with Community Health Agents (CHAs) delivering care to a population within a specific territory. By the PNSB, SUS also provides comprehensive oral health care to the Brazilian population [10, 11]. PNSB represents a transformative strategy focused on expand a comprehensive oral health care in the public health system [12, 13] and it is based on two different strategies.

The first one is increasing the number of oral health teams (OHTs) in Primary Health Care (PHC). OHTs may include a Dental Surgeon and an Oral Health Assistant only; or in addition to these two professionals, an Oral Health Technician. According to the PNSB guidelines, OHTs must work together with a multiprofessional team and be responsible for certain geographic territories and the registered population [9]. The importance of this strategy is based on PHC as the gateway to the system, the guide of care in all dimensions and the provider of the framework for the networks and other services [14].

The second strategy is to achieve comprehensive oral health care in the public health system by expanding the secondary level of oral health care through the Centers of Specialized Dentistry (CEO, *Centros de Especialidades Odontológicas*) [12, 13]. In turn, the second strategy guarantees comprehensive oral health care in the SUS, since CEOs are units that receive patients referenced by OHTs to offer, at a minimum, treatment in the specialties of periodontics, endodontics, patients with special needs, oral diagnosis and minor oral surgery [12, 13].

In this context, national studies on the use of public dental services in the last 5 years have shown that individual and contextual determinants influenced this use, defined as the last dental visit over the last 12 months [5, 15, 16]. However, such studies focus on the concrete use of dental care. There is, therefore, a need to understand the demand for dental care, regardless of its realization or not, since the first focuses on the access achieved by the population and the second intends to answer if some people are more likely to seek dental care [17].

Considering that more than 75% of the adult population need dental care and more than 75% of the population is totally dependent on SUS health care [18, 19], it is also important to identify if some people are more likely to seek dental care in PHC. Mainly because, in addition of being the gateway to the system, this is the ideal level of care for dentistry to use a less invasive and more preventive approach [20].

Moreover, there is a knowledge gap regarding the combination of individual and contextual variables associated with the seek of the offered services. Identifying and analysing these issues is crucial to support the planning and management of dental services, favouring the population’s oral health promotion. Given the above-mentioned facts, the aim of the present study was to evaluate the indicators for dental appointment scheduling in Primary Health Care.

## Methods

### Study design and context

The present is a cross-sectional analytical study reported according to the STrengthening the Reporting of OBservational studies in Epidemiology (STROBE) statement [21]. Our study uses data from the external assessment of the third cycle of the National Program for Improving Access and Quality in Primary Care (PMAQ-AB), carried out between 2017 and 2018, in Brazil.

The PMAQ-AB has been created to improve the quality of public health services by means of strategies for qualification, monitoring and evaluation the health teams’ work in primary health [22–24]. To date, 3 cycles have taken place. Participating municipalities that show an improvement in the standard of quality in care achieve increasing of financial resources transferring. External Assessments was performed with the help of Teaching and Research Institutions from all over the country, by means of interviews with users and professionals of the Health teams. The answers to these interviews constitute the data the 3rd cycle of the multicenter research study [22–24].

### Participants

Microdata obtained from interviews with users and professionals of the Oral Health team from Primary Health Care Units across the country were used in this study, totalling a final sample of 85,231 patients and 22,475 Oral Health teams. The interviews considered in the present study represent the External Assessment of the 3rd cycle of the PMAQ and took place in partnership with Teaching and Research Institutions from all over the country. The supplementary material depicts a detailed presentation of the PMAQ-AB, the used interview instruments and the collection of data.

To access the individuals variables, the interviews were performed with 04 (four) users per team at the Unit. Inclusion criteria: users who did not have an appointment with a doctor or nurse on the day of the interview. Exclusion criteria: users who were going to the health unit for the FIRST time, users who had not been to the unit for more than 12 months and users under 18 years of age. In the present study, users of Health Units that did not have an oral health team, as well as users who did not answer the outcome question were also excluded. Regarding to the variables related to the Oral Health teams, the interviews were performed preferably with the dentist responsible for each team.

### Analysed variables

The analysed variables are presented in Table 1, as well as how the qualitative and quantitative variables were treated in the analysis. Supplementary Material presents the hypotheses and the choice of the variables that were included in this study.

**Table 1:** Outcome and independent variables used in the study

Variable	Presentation
***Outcome***^a^	
To have sought dental appointments in Primary Health Care in the past 12 months	Yes or No It means that the patient has sought dental service in public Primary Health Care in the past 12 months
***Individual independent variables*** **(of patients)**^a^	
Gender	male or female
Age	in full years
Marital status	single, married or in a common-law marriage, divorced, or legally separated, widowed
Self-declared ethnicity	white, black, yellow, brown/mixed-race or indigenous
Education	illiterate, literate, incomplete elementary school, complete elementary school, incomplete high school, complete high school, incomplete higher education, complete higher education, postgraduate
Family income	Total amount in Reais (R$)
Residents in the household.	in numbers
***Contextual independent variables (of the Oral Health teams- OHTs)***^a^	
Total family health teams (FHTs) assisted by an OHT	Number of assisted teams
Frequency at which the OHT attends to users outside the coverage area	Every day, some days of the week, never
The OHT has a territory map	Yes or No
The OHT plans its actions	Yes or No
The OHT participates in meetings together with the FHT	Yes or No
The OHT investigates the epidemiological profile of the population from the territory	Yes or No
The OHT discusses cases and therapeutic projects	Yes or No
The OHT performs a self-assessment process	Yes or No
Self-assessment instrument used	printed AMAQ; electronic AMAQ; AMQ; Instrument developed by the municipality / team; Instrument developed by the State; another assessment tool
Clinical dental file comprises the user’s medical record	Yes or No
The OHT performs actions that are articulated with other social facilities in the territory	Yes or No
The OHT performs scheduled care service	Yes or No
The OHT performs spontaneous search care service	Yes or No
The OHT performs user embracement jointly with the Primary Care team	Yes or No
Main form of scheduling the 1st dental appointment	at the Primary Health Care Unit reception (together with the scheduling for the other professionals); at the dental office (by the oral health team); by the Community Health Agent (ACS); others

*AMAQ* Self-Assessment for Improving Access and Quality of Primary Care (AMAQ, *Auto-Avaliação para a Melhoria do Acesso e da Qualidade da Atenção Básica*), *AMQ* Quality Improvement Assessment (*Avaliação para Melhoria da Qualidade*)

^a^Data source: 3rd Cycle PMAQ-AB [22]

### Statistical methods

The multilevel analysis considered Andersen’s conceptual framework for understanding the multiple dimensions of access to health care [17, 25]. Andersen’s conceptual framework considers that there may be some individuals who are more predisposed to seek health care. Still, there has to be the means, that is, the enabling resources for them to do so. It includes, among other variables, how health care is organized [17, 25].

Initially, the descriptive analyses of individual and contextual variables were performed. For that purpose, absolute and relative frequencies were used for categorical variables, whereas mean, standard deviation, median, minimum and maximum values were used for quantitative variables.

Subsequently, analyses of the associations between the outcome (having sought dental appointments at the Primary Health Care Unit) and individual and contextual variables were performed, using simple and multilevel logistic regression models. The variables in the first level (individual, patients) and in the second level (contextual, Oral Health teams) were considered in the model.

The variables that showed *p* < 0.20 in the crude analyses were studied in the multiple models. Initially, the first-level variables were included in the model, and those with *p* ≤ 0.05 remained after adjustments for the first-level variables. Subsequently, the second-level variables were included, and those with p ≤ 0.05 after the adjustments for the other variables remained in the final model. The gross odds ratio (OR) was estimated by the regression models and adjusted with the 95% confidence intervals. For regression analysis, the variables age, family income and number of people in the family were dichotomized by the median. The adjustment of the models was evaluated by the QIC (Quasi-likelihood Criterion). The analyses were performed using the software R [26].

### Ethical aspects

An Ethics Committee for Research with Human Beings approved the project (CAAE n. 80,477,417.0.0000.0021). The microdata were obtained through public and unrestricted access on June 25, 2019.

## Results

The data of 85,231 patients of 22,475 Oral Health teams were analysed. Table 2 shows the results of the descriptive analyses of the variables at the individual level (patients). Only 58.1% of the patients registered in the Primary Health Care Units with an oral health team had already sought dental appointments. The mean age of patients in the sample is 43.8 years, ranging from 18 to 99 years, with 78.5% of females and 63.4% married or in a common-law marriage.

**Table 2:** Descriptive analysis of the participants’ variables (individual), *n* = 85,231 participants, Brazil 2017

Variable	Category	Frequency (%)
Sought dental care (outcome)	Yes	49,500 (58.1%)
	No	35,731 (41.9%)
Gender	Male	18,360 (21.5%)
	Female	66,871 (78.5%)
Marital status	Single	19,655 (23.1%)
	Married or in a common-law marriage	54,009 (63.4%)
	Divorced/Separated	5677 (6.7%)
	Widowed	5890 (6.9%)
Self-declared ethnicity	White	25,645 (30.1%)
	Black	10,857 (12.7%)
	Yellow	2610 (3.1%)
	Brown/Mixed-race	44,227 (51.9%)
	Indigenous	753 (0.9%)
	No information	1139 (1.3%)
Level of schooling	Illiterate	4818 (5.7%)
	Literate	4525 (5.3%)
	Incomplete Elementary School	27,808 (32.6%)
	Complete Elementary School	8634 (10.1%)
	Incomplete High School	8839 (10.4%)
	Complete High School	22,755 (26.7%)
	Incomplete Higher Education	2977 (3.5%)
	Complete Higher Education	3894 (4.6%)
	Postgraduate Education	858 (1.0%)
	No information	123 (0.1%)
	Mean (standard deviation)	Median (minimum and maximum value)
Age	43.74 (16.31)	42.0 (18.0; 99.0)
Income	1585.58 (2854.39)	980.0 (1.0; 98.500.0)
Number of people in the family	3.60 (1.66)	3.0 (1.0; 30.0)

Table 3 shows the descriptive analyses of the teams’ variables. On average, the teams provide assistance to a mean of 1.2 Family Health teams and 92.8% of the teams attend to people living outside their area of coverage on at least some days of the week. The Self-assessment for the Improvement of Access and Quality of Primary Care (AMAQ) instrument, either in its printed or electronic form, is the main instrument used in the self-assessment process (77.1%). As for the scheduling of patients, this process is carried out at the reception of the Primary Health Care Unit only for 29.3% of the teams, together with the scheduling for the other professionals’ services. Likewise, the clinical dental record is integrated to the user’s medical record in only 76.9% of cases.

**Table 3:** Descriptive analysis of contextual variables (Oral Health Teams) *n* = 22,475 Teams, Brazil 2017

Variable	Category	Frequency (%)
Frequency at which the OHT team attends to users outside the coverage area	Everyday	10,971 (48.8%)
	Some days of the week	9884 (44.0%)
	Never	1620 (7.2%)
The OHT has a territory map	Yes	18,835 (83.8%)
	No	3640 (16.2%)
The OHT plans their actions	Yes	15,111 (67.2%)
	No	7364 (32.8%)
The OHT participates in joint meetings with the FHTs	Yes	17,957 (79.9%)
	No	4518 (20.1%)
The OHT investigates the epidemiological profile of the territory population	Yes	12,436 (55.3%)
	No	10,039 (44.7%)
The OHT discusses cases and therapeutic projects	Yes	10,710 (47.7%)
	No	11,765 (52.3%)
The OHT performs the self-assessment process	Yes	18,175 (80.9%)
	No	4300 (19.1%)
Self-assessment instrument used	Printed AMAQ	11,277 (50.2%)
	Electronic AMAQ	6045 (26.9%)
	AMQ	105 (0.5%)
	Municipality Instrument	396 (1.8%)
	State Instrument	73 (0.3%)
	Other instruments	279 (1.2%)
	Not applicable	4300 (19.1%)
The clinical dental file is integrated with the user’s medical record	Yes	17,277 (76.9%)
	No	5198 (23.1%)
The OHT performs actions articulated with other social facilities in the territory	Yes	20,570 (91.5%)
	No	1905 (8.5%)
The OHT performs scheduled service	Yes	22,036 (98.0%)
	No	439 (2.0%)
The OHT performs spontaneous search service	Yes	22,040 (98.1%)
	No	435 (1.9%)
The OHT performs user embracement jointly with the FHT	Yes	18,674 (83.1%)
	No	3714 (16.5%)
	Not applicable	87 (0.4%)
Main type of scheduling the 1st dental appointment	At the BHU reception	6585 (29.3%)
	At the dental office	11,661 (51.9%)
	Through CHAs	3868 (17.2%)
	Another type	361 (1.6%)
**Variable**	**Mean (standard deviation)**	**Median (minimum and maximum value)**
Number of assisted OHTs	1.22 (0.67)	1.0 (1.0; 9.0)

It is also observed that 79.9% of the teams participate in meetings together with the Primary Care team, but only 47.7% carry out the discussion of cases and therapeutic projects. The epidemiological profile of the assisted population is investigated by 55.3% of the interviewed teams. In turn, most teams (91.5%) carry out actions that are integrated with other social facilities in the territory and user embracement is performed jointly with the Primary Care team (83.1%).

In the crude analyses, where each variable was analysed individually, all variables showed a significant association with the outcome “having sought dental care with the dentist in this health Primary Health Care Unit” (*p* < 0.05). In the final model, after the adjustment of variables, the independent variables that showed significant associations with the outcome variable were gender, age, ethnicity/skin colour, level of schooling, income and number of people in the family at the patient level and number of Family Health Teams (FHTs) to which the OHTs provide assistance, frequency with which it attends to users outside the coverage area, whether it participates in meetings together with the FHT, if it investigates the epidemiological profile of the territory population, if there is a discussion of cases and therapeutic projects, if the clinical dental file is part of the user’s medical record, if the OHT carries out actions with other social facilities in the territory and if the OHT professionals carry out user embracement together with the FHT at the OHT level (Table 4).

**Table 4:** Results of the multilevel logistic regression models for the outcome “seeking for dental appointment scheduling in Primary Health Care Unit” (*n* = 85,231 participants from 22,475 teams), Brazil, 2017

Variables	Crude Analyses				Final multilevel model			
	Coefficient	Standard error	Crude OR 95% CI)	*p*-value	Coefficient	Standard error	Adjusted OR (95% CI)	*p*-value
**Individual level**								
Gender								
Female (Ref. = male)	0.32	0.02	1.38 (1.34–1.43)	< 0.0001	0.11	0.02	1.12 (1.07–1.16)	< 0.0001
Age								
Up to 42 years (Ref. > 42 years)	0.86	0.01	2.37 (2.31–2.44)	< 0.0001	0.71	0.02	2.03 (1.96–2.10)	< 0.0001
Marital status								
Single (Ref. = married)	0.18	0.02	1.20 (1.16–1.24)	< 0.0001	–	–	–	–
Separated (Ref. = married)	−0.30	0.03	0.74 (0.70–0.78)	< 0.0001	–	–	–	–
Widowed (Ref. = married)	− 0.82	0.03	0.44 (0.42–0.47)	< 0.0001	–	–	–	–
Self-declared ethnicity								
Black (Ref. = white)	0.20	0.02	1.23 (1.17–1.29)	< 0.0001	0.12	0.03	1.12 (1.07–1.19)	< 0.0001
Yellow (Ref. = white)	0.22	0.04	1.25 (1.15–1.36)	< 0.0001	0.03	0.05	1.03 (0.94–1.13)	0.5740
Brown (Ref. = white)	0.25	0.02	1.29 (1.25–1.33)	< 0.0001	0.09	0.02	1.10 (1.06–1.14)	< 0.0001
Indigenous (Ref. = white)	0.33	0.08	1.39 (1.20–1.61)	< 0.0001	0.19	0.09	1.21 (1.02–1.44)	0.0317
Level of schooling (Ref. Complete Elementary School)	0,37	0.01	1.44 (1.40–1.48)	< 0.0001	0.11	0.02	1.12 (1.08–1.16)	< 0.0001
Family income (Ref. > R$980)	0,32	0.02	1.37 (1.33–1.42)	< 0.0001	0.24	0.02	1.27 (1.23–1.31)	< 0.0001
People in the family (Ref. ≤3)	0,46	0.01	1.58 (1.54–1.63)	< 0.0001	0.33	0.02	1.39 (1.35–1.44)	< 0.0001
**Contextual level (Teams)**								
Number of assisted FHTs (Ref. > 1)	0.34	0.02	1.40 (1.36–1.46)	< 0.0001	0.25	0.03	1.29 (1.23–1.36)	< 0.0001
Frequency at which the OHT attends to users outside the area								
Some days (Ref. = daily)	0.08	0.01	1.09 (1.06–1.12)	< 0.0001	0.07	0.02	1.07 (1.03–1.11)	0.0004
Never (Ref. = daily)	0.06	0.03	1.06 (1.00–1.12)	0.0412	0.10	0.04	1.11 (1.03–1.19)	0.0067
Territory map (Ref. = no)	0.19	0.02	1.21 (1.16–1.25)	< 0.0001	–	–	–	–
Planning of actions (Ref. = no)	0.16	0.01	1.17 (1.14–1.20)	< 0.0001	–	–	–	–
Meetings together with the FHT (Ref. = no)	0.25	0.02	1.28 (1.24–1.32)	< 0.0001	0.12	0.02	1.13 (1.08–1.19)	< 0.0001
Investigation of the epidemiological profile (Ref. = no)	0.19	0.01	1.21 (1.18–1.24)	< 0.0001	0.11	0.02	1.12 (1.07–1.16)	< 0.0001
Disses cases and therapeutic projects (Ref. = no)	0.15	0.01	1.16 (1.13–1.19)	< 0.0001	0.08	0.02	1.08 (1.04–1.13)	0.0003
Self-evaluation process (Ref. = no)	0.18	0.02	1.20 (1.16–1.25)	< 0.0001	–	–	–	–
Instrument used								
Printed AMAQ (Ref. = electronic AMAQ)	0.10	0.02	1.11 (1.07–1.14)	< 0.0001	–	–	–	–
AMQ (Ref. = electronic AMAQ)	−0.00	0.10	1.00 (0.82–1.22)	0.9963	–	–	–	–
Municipality instrument (Ref. = electronic AMAQ)	−0.12	0.05	0.89 (0.80–0.98)	0.0257	–	–	–	–
State instrument (Ref. = electronic AMAQ)	−0.24	0.12	0.79 (0.62–1.00)	0.0538	–	–	–	–
Another instrument (Ref. = electronic AMAQ)	−0.08	0.06	0.93 (0.82–1.05)	0.2283	–	–	–	–
Clinical dental file integrated with medical record (Ref. = no)	0,26	0.02	1.29 (1.25–1.33)	< 0.0001	0.19	0.02	1.21 (1.15–1.26)	< 0.0001
Articulated actions (Ref. = no)	0,21	0.02	1.23 (1.17–1.29)	< 0.0001	0.09	0.03	1.10 (1.03–1.18)	0.0068
Programmed care (Ref. = no)	0,16	0.05	1.17 (1.06–1.29)	0.0013	–	–	–	–
Attends to spontaneous search (Ref. = no)	0,15	0.05	1.16 (1.05–1.28)	0.0033	–	–	–	–
Joint user embracement (Ref. = no)	0,28	0.02	1.32 (1.27–1.37)	< 0.0001	0.14	0.02	1.15 (1.10–1.21)	< 0.0001
Scheduling								
At the reception (Ref. = at the office)	0,13	0,02	1,14 (1,11-1,18)	< 0,0001	–	–	–	–
Through the CHA (Ref. = at the office)	0,25	0,02	1,28 (1,23-1,33)	< 0,0001	–	–	–	–
Another type (Ref. = at the office)	−0,20	0,05	0,82 (0,74-0,91)	0,0003	–	–	–	–
Akaike Information Criterion (AICc)					90.952,17		90.146.69	

*QIC* Quasi-likelihood Criterion (empty model): 115.923,22. *OR* Odds ratio, *CI* confidence interval

The chance of seeking dental care from the dentist at the Primary Health Care Unit is significantly higher among women, younger patients, patients of black, brown, and indigenous ethnicity compared to white patients, and among patients with a higher level of education, with lower income and larger families.

In turn, the search for dental appointments is significantly higher in the teams that attend to a single FHT, in those with a lower frequency of care provided to people living outside the coverage area, who participate in meetings together with the FHT, which investigate the epidemiological profile of the assisted population, who discuss cases and therapeutic projects, in which the clinical dental file is included in the user’s medical record, which performs actions articulated with other social facilities in the territory and which perform user embracement together with the Primary Care team.

Among the variables of the individual level, the patient’s age (OR = 2.03, 95% CI: 1.96–2.10) was the one with the greatest effect on the outcome, whereas among the teams’ variables, working with only one Primary Care team (OR = 1.29, 95% CI: 1.23–1.36) and having the clinical dental file included in the user’s medical record (OR = 1.21, 95% CI: 1.15–1.26) were the variables with the greatest effect.

## Discussion

This study showed that the seeking for dental appointments in Primary Health Care is related to variables of individuals and also of the organization of oral health teams; additionally, it showed that only 58.1% of the users interviewed at these Primary Health Care Units seek the available dental care. With a pioneering and bold approach in comparison with other public health systems worldwide, Brazil offers its citizens universal oral health care services, from prevention to rehabilitation. By taking on the task of evaluating the indicators of the search for dental appointments in Primary Health Care Units with an Oral Health Team, this study aims to bring a contribution to oral health management, instrumentalizing the fight for the reduction of inequalities regarding the access to oral health in Brazil.

Therefore, it is evident that despite the historical debt the Brazilian government has with its citizens regarding the offer of universal oral health services, the search for these services – currently, finally offered to all age groups of the population – remains modest. There is an epidemiologically recorded accumulation of oral health needs in the country. For instance, the latest national oral health survey disclosed that 75.2% of the adult population (aged 35 to 44 years) needed dental care in Brazil [18]. However, the services offered at the PHC level are sought after, on average, by slightly more than 50% of the citizens of the territory, even when more than 75% of the Brazilian population is totally dependent on SUS health care [19].

As for individual indicators, our study showed that the seek for dental care is greater among younger patients, a result that has been explained, since edentulism is a predominant condition among elderly Brazilians [3]. Maintaining the idea of dental care focused on tooth care discourages the elderly from performing oral health self-care. Our study also showed that people living in a more crowded household are more likely to seek dental appointments. This result can be explained by the importance of the family in health care, in the search for information, in the search for a diagnosis, in the construction of therapeutic itineraries, in daily, permanent and long-term care [27].

The fact that men seek health services less frequently than women, even if they have serious health problems, is well established in the literature [28, 29], and such behaviour is attributed to cultural issues. In turn, the greater demand for dental care among black, brown and indigenous patients in comparison to white patients can be understood, as dental treatment needs also reflect racial inequalities, with worse indicators of oral health (caries, tooth loss, pain and need for prosthesis) being observed in the black and indigenous population [3, 30].

Regarding the contextual indicators, considering the results found in our study, we could understand that the seeking for dental care is directly related to a better interaction and the articulation of the oral health team with the other members of the Primary Care Health team and with the population of the assisted territory. In 2004, through the *Brasil Sorridente* program, a series of policies were implemented aiming at the expansion and qualification of oral health in PHC [2, 11]. The program highlights the importance of knowing the reality of the covered areas and the building of problem-solving and effective practices regarding the care offered by the OHT.

The inclusion of the Oral Health Team in the Family Health Strategy brought as the directive the performance of actions in an integrated perspective of health prevention, promotion and provision of care. *Brasil Sorridente* underlines that OHTs must have knowledge of the territory, be close to those assisted and joint planning and participation of the entire team to assist the population [31]. However, despite all the attained advances, oral health access difficulties still persist in SUS. The challenges faced by the oral health teams are related to the comprehensive care, expansion and qualification of assistance, teamwork, planning, monitoring and evaluation of actions and working conditions [2].

The reasons for the persistence of these difficulties can be identified when the results of our study show that the Oral Health Teams provide assistance, on average, to 1.2 PHC teams and the vast majority attend to individuals living outside the covered area on at least some days of the week. Such findings reflect the failure to match the number of OHTs affiliated to family health teams (FHTs) as recommended by the *Brasil Sorridente* guidelines. This situation becomes even more serious in the face of changes in the Brazilian political and economic framework in recent years. Constitutional Amendment number 95 of 2016 imposed a severe withdrawal of investments in oral health in SUS [32].

In the wake of the political-institutional decisions that Brazil made as of 2016, the new National Primary Care Policy (PNAB, *Política Nacional de Atenção Básica*) published in 2017 sees oral health as non-mandatory in the Family Health Strategy. PNAB has already reflected in an increase in the number of municipalities that reduced the number of oral health teams in the Family Health Strategy [11, 33]. It is already possible to detect loss of implantation amplitude, indicated by the stabilization trend line regarding the number of these teams after January 2018. Between the 1st and the 21st months of the publication of the PNAB 2017 Ordinance, a three-fold increase was observed regarding the number of municipalities that reduced the number of OHTs in the Family Health Strategy [11]. Obviously, the direct negative impact of this fact on citizens’ access to oral health services should be more clearly perceived in the coming years.

The concept of Primary Health Care, the field in which the present investigation was developed, demonstrates that, in essence, PHC is aimed at caring for people, not diseases. Therefore, it has to be accessible, comprehensive, continuous and guarantee the coordination of care for cases that require referrals [34, 35]. This explains the fact that the present study has demonstrated that the user’s chance to seek dental care is greater in OHTs that discuss cases and therapeutic projects. It is evident the need to rethink health work processes, changing the focus of attention, currently on the disease, so that it is focused on the individual and the community. This is not merely about improving the quality of care (albeit it is) but, above all, it is about the observance of Integrality in health, a fundamental SUS principle.

From the perspective of overcoming the challenges that citizens meet to have access to dental care in the context of SUS, we point out the determining role of Community Health Agents (CHAs) regarding the relationship between the community and health services. The present investigation showed that the search for dental appointments was significantly higher in OHTs that carry out actions articulated with other social facilities in the territory and that perform user embracement together with the Primary Care team. Worldwide, CHAs are seen as an important link to increase the access of communities to services, especially for people living in areas of greater vulnerability and, thus, to accelerate and sustain the progress in meeting health targets in Sustainable Development Goals (SDGs) [36, 37].

The strengths of this research comprise the sample size and distribution, which included participants from all over the national territory of a country known for its continental dimensions. In turn, we point out possible limitations of our study. A cross-sectional design does not allow the identification of causal relationships and the absence of questions related to behaviour or about the needs perceived by users, variables that were not collected in the PMAQ.

 In this sense, further research, particularly with a qualitative analysis, can contribute to the consolidation of scientific knowledge. Furthermore, it is important to emphasize that in Brazil health care can be provided by the public health system and also by the private system, through direct payment from the patient to the service provider.

It should be clarified that health financing systems can have a different impact on the patient’s decision-making regarding the search for dental care [38]. Our study used information related only to public financing of health care and this can be pointed out as a limitation of the research. Another limitation is that the questionnaire did not ask whether users who did not seek to schedule an appointment in the public service did so in the private service. However, it is estimated that the SUS is exclusively responsible for assisting more than 75% of the Brazilian population [19]. Percentage for dental care needs can be even higher.

Based on the findings of the present study, we suggest the need for strengthening oral health teams in the Unified Health System and for following guidelines proposed by the *Brasil Sorridente* program. Policies that reinforce the need for articulation among oral health teams and other Primary Care members, as well territory, will contribute to the improvement of access to services and to the oral health epidemiological profile of the assisted population. In addition, it can be observed that individual indicators reflect the oral health inequalities that still persist in the Brazilian population. These inequalities can be fought with greater investment in public oral health policies.

## Conclusion

It is concluded that individual issues that do not favour the search for oral health care, such as being older than 42 years old, need to be considered both by the oral health team, as well as by the managers who organize and plan the services. In turn, the users most often seek after oral health teams that effectively put the *Brasil Sorridente* guidelines into practice, such as assisting only one FHT. Thus, equating the number of oral health teams and primary health care teams should be encouraged by health services mangers.

## Supplementary Information

**Additional file 1.** PMAQ information.

## Data Availability

Data analysis has not yet been exhausted. For information on accessing the data, please contact the corresponding author.

## Abbreviations

AMAQ: Self-assessment for the Improvement of Access and Quality of Primary Care (*Auto-avaliação para a Melhoria do Acesso e da Qualidade da Atenção Básica*)
AMQ: Evaluation for Quality Improvement (*Avaliação para Melhoria da Qualidade*)
CHA: Community Health Agent
FHT: Family health team
OHT: Oral health team
PMAQ-AB: National Program for Improving Access and Quality in Primary Care (*Programa Nacional de Melhoria do Acesso e da Qualidade da Atenção Básica*)
PNAB: National Primary Care Policy (*Política Nacional de Atenção Básica*)
PNSB: National Oral Health Policy (*Política Nacional de Saúde Bucal*)
SUS: Unified Health System (*Sistema Único de Saúde*)
