# Supplementary material for: Indicators for dental appointment scheduling in primary health care: a national cross-sectional study
Source: BMC Public Health. 2021 Dec 8;21:2234. doi: 10.1186/s12889-021-12319-x (PMC8656053; doi:10.1186/s12889-021-12319-x)
Supplement: Supplementary file 1 — Additional file 1. PMAQ information. [file 12889_2021_12319_MOESM1_ESM.docx]

**Indicators for dental appointment scheduling in Primary Health Care: a national cross-sectional study**

Estêvão Azevedo Melo^1^, Livia Fernandes Probst^1^, Luciane Miranda Guerra^1^, Elaine Pereira da Silva Tagliaferro^2^, Alessandro Diogo De-Carli^3^, Antonio Carlos Pereira^1*^

**Supplementary Material**

**Additional file 1.** PMAQ information.

This file presents detailed presentation of data collection from the External Assessment (EA) of the 3^rd^ Cycle of the National Program for Improving Access and Quality in Primary Care (PMAQ). All information described here is available to the public through the link: <https://aps.saude.gov.br/ape/pmaq/ciclo3/>.

The PMAQ-AB, created in 2011, depicts a Cross-sectional Strategic Development axis, which comprises a continuous cycle of Primary Care (PC) access and quality improvement. In 2017, the External Assessment of the 3^rd^ cycle of the PMAQ took place in partnership with the Teaching and Research Institutions (TRIs) from all over the country, with acknowledged experience in evaluation research in the organization and development of field work. The partner TRIs were responsible for selecting and training the field work teams, consisting of supervisors and interviewers [1–3].

In this context, we present the data collection instrument for external assessment, in which a set of actions was carried out to investigate the conditions of access and quality of all municipalities and primary care teams participating in the program. The aim is to recognize and value the efforts and results of teams and municipal health managers in Primary Care qualification.

The external assessment instrument for the primary care and oral health teams is organized into six modules, according to the information collection method:

• Module I - Basic Health Unit surveillance. It aims to assess the conditions of the Basic Health Unit infrastructure, materials, supplies and medications.

• Module II - Interview with the Primary Care Team professional and verification of documents in the Basic Health Unit. It aims to evaluate the team’s work process and the organization of the service and user care.

• Module III - Interview with 04 (four) users per team at the Basic Health Unit (Basic Health Unit User Care Satisfaction Survey in Brazil). It aims to verify the users’ satisfaction and perception regarding health services related to their access and use.

• Module IV - Interview with the professional from the Family Health Support Center (NASF, *Núcleo de Apoio à Saúde da Família*) and verification of documents in the Basic Health Unit. It aims to evaluate these teams’ work processes and the organization of care for the users.

• Module V - Basic Health Unit surveillance regarding Oral Health aiming to assess the conditions of infrastructure, materials, and dental care supplies.

• Module VI - Interview with the Oral Health Team professional and verification of documents in the Basic Health Unit. It aims to evaluate the oral health work process and the organization of the service and care for the users.

In total, data from 38,865 family health teams, 25,090 oral health teams, 4,110 Extended Family Health and Primary Care Centers, 30,346 Family Health Units and 140,444 users were made available. Data from Modules III and VI were used for the purposes of this study.

***Module III instrument***: refers to a questionnaire that was applied to four users per team present at the unit on the day of the external assessment. The users were approached by the interviewers at the Unit and the interview took place if the previously established eligibility criteria were met.

Inclusion criteria: users who did not have an appointment with a doctor or nurse on the day of the interview. Exclusion criteria: users who were going to the health unit for the FIRST time, users who had not been to the unit for more than 12 months and users under 18 years of age. In the present study, users of Health Units that did not have an oral health team, as well as users who did not answer the outcome question were also excluded.

***Module VI Instrument:*** Interview with a professional from the Oral Health Team and verification of documents confirming the answers at the Health Unit. To conduct the interview, prior contact was made with the professional. The professional responsible for answering the questions was the dentist (DS). If the DS were absent at the time of the external assessment, another professional from the oral health team (Oral Health Assistant or Oral Health Technician) could be interviewed. Regarding the questions that involved document verification, the interviewed professional indicated in the said document the elements that confirmed the question, aiming to facilitate the verification process.

At the end, we obtained a sample of 85,231 patients and 22,475 Oral Health teams, as shown below.


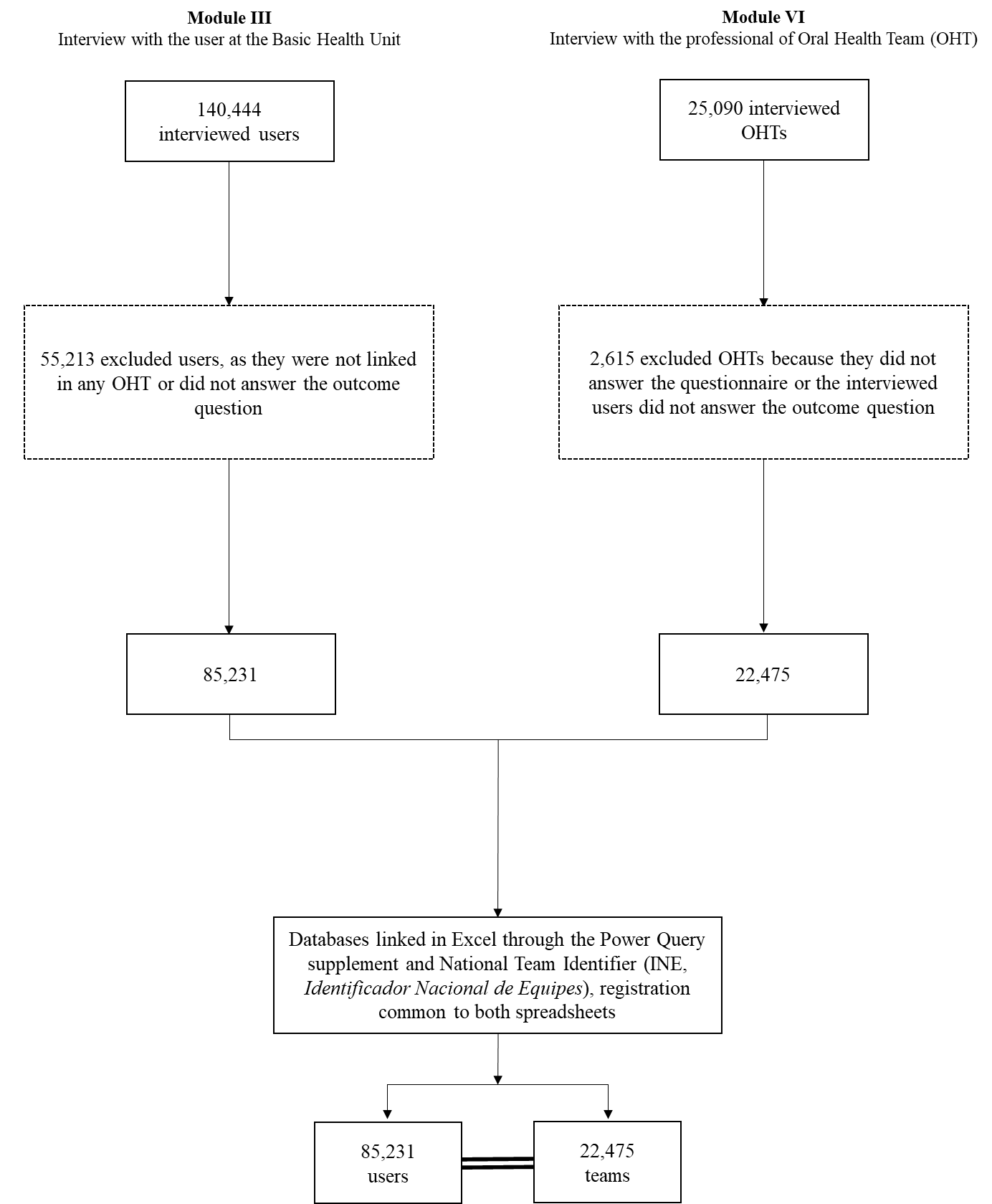


**Figure S1. Database organization.**

The choice of the analyzed variables will be explained clearly below:

***1. Definition of the outcome variable***: question 18.1 of the users’ questionnaire contained the following statement: “Can you make an appointment with the dentist at this health unit?”; and the following options for recording by the interviewer: -Yes, -No -Never sought an appointment with a dentist in this Unit, -This unit does not have an oral health team or -Does not know / did not answer.

As the aim of our study was to evaluate the user's relationship with the oral health team, we started by excluding those that checked the last two options. Subsequently, we observed a high percentage of users who reported never having sought dental care. Based on this observation, we elected and categorized the dependent variable as follows:

- The user has previously sought dental care in this Unit: ‘Yes’, they have already sought, regardless of whether they were able to schedule an appointment or not, that is, we grouped the yes or no answers to question 18.1 of the Module III instrument; and ‘No’, never sought an appointment with the dentist at this Unit, so that we could identify the predictors of the demand for care.

The choice of individual and contextual variables was based on the explanatory hypotheses presented below and supported by the Andersen's conceptual framework for understanding the multiple dimensions of access to health care [4, 5].

***2. Definition of individual variables***: The choice of individual variables considers that there may be some individuals who are more predisposed to seek for oral care [4, 5]. Thus, still in the same instrument we have chosen questions related to economic and sociodemographic variables, corresponding to item 3-Identification:

• Gender

•Marital status

• Self-declared ethnicity

• Level of schooling

•Age

•Income

• Number of people in the family

***3. Definition of contextual variables*** (related to the OHT):

Andersen's conceptual framework recognizes that even though some individuals are more predisposed to seek care, there has to be the means, that is, the enabling resources for them to do so [4]. The conceptual framework considers that the contextual level includes how physical and human resources are structured to offer health services. It includes, among other variables, the proportion of health professionals and how care is organized [5].

Therefore, for the contextual variables, we selected in the Module VI instrument the questions that could hypothetically hinder or facilitate the team's work process, as well as the creation of a link with the user:

*Questions in item 3 - Territorialization and Reference Population of the Oral Health Team:*

• Number of assisted FHTs

• Frequency that OHT attends to users outside the coverage area

• OHT has a map of the territory

The greater the number of FHTs assisted by an OHT and the greater the frequency of assistance to users outside the coverage area, more time is hypothetically used only in clinical care assistance, making it more difficult to create a link with patients. Moreover, it was hypothesized that the territory map can be used as a tool that facilitates the team work and identifies the needs in the area. For the question related to the territory map, they were asked to present document evidence and, therefore, we considered as ‘Yes” the teams that answered affirmatively and also presented a document that confirmed the answer.

*Questions in item 4 - Planning, Follow-up and Evaluation:*

• OHT plans their actions

• OHT participate in meetings together with the FHT

• OHT investigates the epidemiological profile of the territory population

• OHT discusses cases and therapeutic projects

• OHT performs a self-assessment process

• Self-assessment instrument used

Hypothetically, the more articulated regarding the planning, epidemiological reality of the territory and the more integrated with teamwork, participating in joint meetings and carrying out discussion of cases and therapeutic projects, the more prepared the OHT will be to identify patients in need of the service or even those who, although they need it, do not seek the service spontaneously. Moreover, hypothetically, the self-assessment process can help the team reflect on their actions and readjust those they consider opportune and feasible in the local context. Document evidence was requested for all questions in this item and, therefore, we considered as 'Yes' the teams that answered the question affirmatively and also showed document evidence for the answer.

*Questions in item 6 - Organization of Medical Records in the Health Unit:*

• Clinical dental file is included in the user's medical record

Oral health must be considered as inextricable from overall health. Thus, hypothetically, units that work with a single medical record and not separated by areas, as health care is focused on the individual and not only on the disease, which can facilitate the link between users and teams. Document evidence was requested for this question, and, therefore, we considered as ‘Yes” the teams that answered affirmatively and also showed document evidence for the answer.

*Questions in item 7 - Organization of the Agenda and Offer of Team Actions:*

• OHT carries out actions articulated with other social facilities in the territory

• OHT performs scheduled service

• OHT performs spontaneous demand service

• OHT performs user embracement jointly with the FHT

• Main type of scheduling the 1^st^ dental appointment

Hypothetically, the more integrated with the set of professionals at the Unit and the social facilities of the territory, the more opportune will be for patients to recognize the importance of the performance and service provided by OHT. Finally, the more diverse the forms of care, including the spontaneous and the programmed forms, it is hypothesized that the easier will be for patients to obtain the care and, therefore, seek it.

**References**

1. Brasil. Ministério da Saúde. Instrumento de avaliação externa do saúde mais perto de você - acesso e qualidade. Programa Nacional de Melhoria do Acesso e da Qualidade da Atenção Básica (PMAQ). 2017. http://189.28.128.100/dab/docs/portaldab/documentos/instrumento_amaq_ab_sb_3ciclo.pdf.

2. Pinto HA, Sousa ANA de, Ferla AA. O Programa Nacional de Melhoria do Acesso e da Qualidade da Atenção Básica: faces de uma política inovadora. Saúde em Debate. 2014;38 special:358–72.

3. Macinko J, Harris MJ, Rocha MG. Brazil’s National Program for Improving Primary Care Access and Quality (PMAQ). J Ambul Care Manage. 2017;40:S4–11.

4. Baker SR. Applying Andersen’s behavioural model to oral health: what are the contextual factors shaping perceived oral health outcomes? Community Dent Oral Epidemiol. 2009;37:485–94. doi:10.1111/j.1600-0528.2009.00495.x.

5. Andersen RM, Davidson PL. Improving Access to care in America: Individual and Contextual Indicators. In: RM A, TH R, GF K, editors. Changing the U.S. health care system: Key issues in health services policy and management. 3rd edn. San Francisco: Jossey-Bass; 2007. p. 3–31.
